# Magnetic Biochar Derived from Fenton Sludge/CMC for High-Efficiency Removal of Pb(II): Synthesis, Application, and Mechanism

**DOI:** 10.3390/molecules28134983

**Published:** 2023-06-25

**Authors:** Zongwu Wang, Juan Guo, Junwei Jia, Wei Liu, Xinding Yao, Jinglan Feng, Shuying Dong, Jianhui Sun

**Affiliations:** 1 Department of Environment Engineering, Yellow River Conservancy Technical Institute, Kaifeng Engineering Research Center for Municipal Wastewater Treatment, Kaifeng 475004, China; 2 MOE Key Laboratory of Yellow River and Huai River Water Environmental and Pollution Control, School of Environment, Henan Normal University, Xinxiang 453007, China

**Keywords:** magnetic biochar, Fenton sludge, Pb(II) removal, adsorbent

## Abstract

Magnetic biochar composites (MBC) were developed by a simple one-step pyrolysis method using Fenton sludge waste solid and carboxymethyl cellulose sodium. Detailed morphological, chemical, and magnetic characterizations corroborate the successful fabrication of MBC. Batch adsorption experiments show that the synthesized MBC owns high-efficiency removal of Pb(II), accompanied by ease-of-separation from aqueous solution using magnetic field. The experiment shows that the equilibrium adsorption capacity of MBC for Pb(II) can reach 199.9 mg g^−1^, corresponding to a removal rate of 99.9%, and the maximum adsorption capacity (*q_m_*) reaches 570.7 mg g^−1^, which is significantly better than that of the recently reported magnetic similar materials. The adsorption of Pb(II) by MBC complies with the pseudo second-order equation and Langmuir isotherm model, and the adsorption is a spontaneous, endothermic chemical process. Investigations on the adsorption mechanism show that the combination of Pb(II) with the oxygen-containing functional groups (carboxyl, hydroxyl, etc.) on biochar with a higher specific surface area are the decisive factors. The merits of reusing solid waste resource, namely excellent selectivity, easy separation, and simple preparation make the MBC a promising candidate of Pb(II) purifier.

## 1. Introduction

Motivated by toxic threats of Pb(II) toward the human central nervous system and genital system, a wealth of remediation techniques have been employed to remove Pb(II) from contaminated water, and adsorption stands out for its ease-of-operation, flexible design, and high effectiveness. During the past few decades, diverse efforts have been devoted to fabricating reliable engineered materials, and biochar (BC) obtained from various biomass by thermal decomposition are becoming the boosting issue in the fields owing to its large area, high porous structure, and rich oxygen-containing functional groups (e.g., hydroxyl, carboxyl, and phenolic groups) [1,2]. Heavy metal adsorbents using agricultural and forestry waste such as leaves, rice husks, straw, sawdust, rice straw, and manure pellet as carbon sources [1,3,4,5,6], and heavy metal adsorbents using monosaccharide glucose as precursor using a hydrothermal method [7,8], and biochar (BC) fixed on zero valent iron particles using polysaccharides starch and activated sludge as carbon sources for the removal of Cr(VI) and Pb(II) from water all have been prepared successfully [9]. Furthermore, considering the practical difficulty of separating and recovering of the spent powder biochar from aqueous solution, magnetic biochar composites (MBC) maintaining the outstanding characteristics of biochar has received extensive attention for its ease separation [10,11,12]. Whereas relatively higher cost of raw materials seriously limits the industry-level use of MBC.

Fenton sludge (FS) is produced during Fenton and Fenton-like oxidation processes during the treatment of waste water, and is defined as a hazardous solid waste for containing large amount of iron ions, organic matter, microorganisms, humic acids, some toxic and harmful bacteria, heavy metals, organic pollutants, etc., and must be disposed by professional company. Moreover, Fenton sludge can be used as effective magnetic source of MBC, converting industrial solid waste to highly valuable magnetic materials. Direct reuse of this raw-material integration will largely simplify the preparation process and reduce the cost of MBC. Therefore, the research on safe and green utilization of Fenton sludge has witnessed growing attention [13]. Magnetic biochar composites (MBC) derived from biological sludge and Fenton sludge have been prepared by hydrothermal carbonization, and can serve as effective catalysts for heterogeneous catalysis degradation of dyeing wastewater and aniline pollutants [14,15]. MBC was synthesized by hydrothermal method using Fenton iron sludge as a raw material, and can be used as skeleton materials to promote dewatering of sewage sludge for the promotive effect on decomposition of protein [13,16]. As a kind of common polysaccharide substance, sodium carboxymethyl cellulose (CMC) possesses the advantages of sustainable resources and inexpensive cost in the meantime, and belongs to a promising precursor of MBC. To the best of our knowledge, the conversion of Fenton sludge solid waste and CMC to MBC targeting of heavy metal removal from the environment has been rarely reported. Our work will promote the 3Rs (reduce, reuse, recycle) of solid waste resource and large-scale use of MBC.

Considering the foregoing analysis on the current research gaps, we herein constructed an emerging magnetic biochar composites (MBC) by incorporating Fenton sludge (FS) solid waste and sodium carboxymethyl cellulose (CMC) to solve the removal of Pb(II). Both morphological, chemical, and magnetic characterizations, and batch adsorption experiments were conducted. The objectives of this research were the following: (1) to fabricate MBC by one-step pyrolysis method targeting the 3R of Fenton sludge solid waste and the effective capture of Pb(II) ions; (2) to investigate in detail the adsorption performance of Pb(II) on MBC through routine experiments; (3) to reveal the plausible adsorption mechanism by the FT-IR and XPS technologies; and (4) to verify the great application prospects of MBC in Pb(II)-contaminated wastewater.

## 2. Results and Discussion

### 2.1. Materials and Characterization

**Figure 1:**
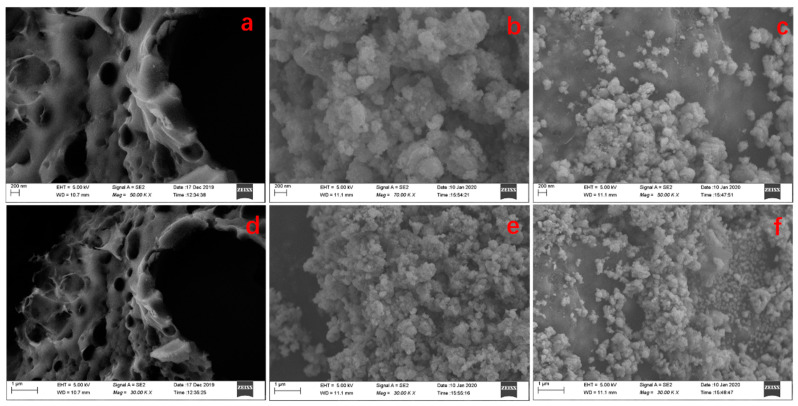
SEM images of CMC-450 (**a**,**d**), FS-450 (**b**,**e**), and MBC (**c**,**f**).

The morphologies of MBC, CMC-450, and FS-450 were observed by scanning electron microscope (SEM), as shown in Figure 1. Obviously the CMC-450 illustrates a honeycomb-like pore structure with a pore size range of 200–600 nm (Figure 1a,d), which can provide a relatively large specific surface area and contribute to a more efficient adsorption. The FS-450 is the mixture of uniform iron oxide nanoparticles with a diameter of 100~300 nm (Figure 1b,e), and large specific surface area also helps to improve adsorption efficiency. Compared to the CMC-450 and the FS-450, upon the decoration with nanoparticles, ample iron oxide embedded on the surface of the thin layer carbon matrix in MBC (Figure 1c,f), which not only ensures the uniform distribution of magnetism, but also increases the specific surface area of MBC.

**Figure 2:**
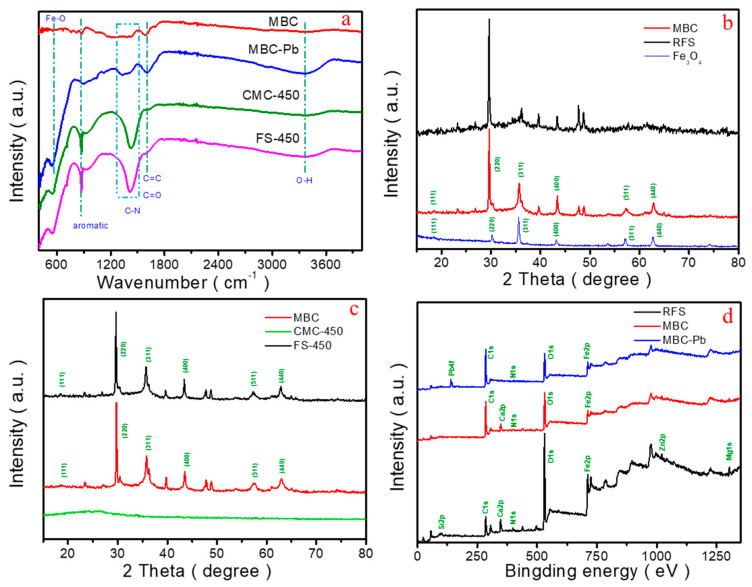
The FT−IR (**a**), XRD (**b**,**c**), and XPS (**d**) patterns of as-prepared samples.

To further probe the chemical constitutions of RFS, CMC-450, and MBC, The Fourier transform infrared (FT-IR), X-ray diffraction (XRD) and X-ray photoelectron spectroscopy (XPS) were performed (Figure 2). From the FT-IR spectra of MBC (Figure 2a), characteristic peaks located at 3300, 1660, and 1415 cm^−1^ represented the O-H, C=O/C=C and aromatic functional groups, respectively [13,17], which provide the possibility of efficient adsorption toward heavy metals by MBC. Furthermore, the peak of 570 cm^−1^ is ascribed to the presence of Fe-O in MBC, indicating the existence of Fe_3_O_4_ components, which also can be verified by XRD analysis subsequently (Figure 2b). Compared to RFS, the diffraction peaks of MBC correspond well with the (111), (220), (311), (400), (511), and (440) reflections at 2θ = 18.3°, 30.1°, 35.4°, 43.1°, 53.4°, 56.9°, and 62.5°, revealing the formation of Fe_3_O_4_ phase (JCPDS No.19-0629) [18]. Figure 2c shows that both FS-450 and MBC exhibit XRD peak signals of Fe_3_O_4_, indicating the existence of magnetism. That is consistent with the FT-IR test results, completely different from CMC-450.

**Figure 3:**
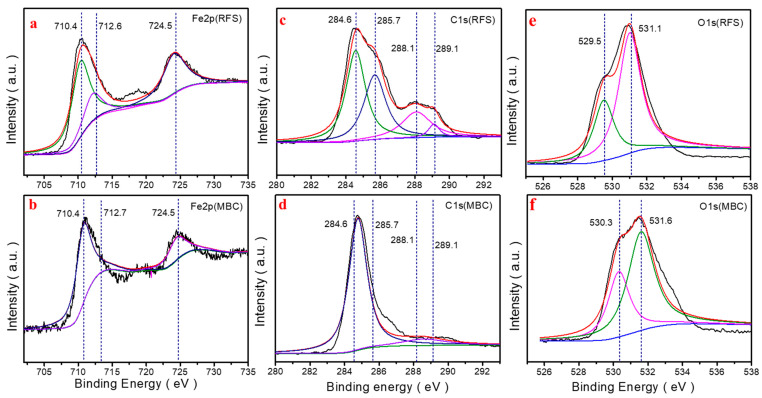
High-resolution XPS spectra of (**a**) Fe_2p_ (RFS), (**b**) Fe_2p_ (MBC), (**c**) C_1s_ (RFS), (**d**) C_1s_ (MBC), (**e**) O_1s_ (RFS), (**f**) O_1s_ (MBC) for the samples.

Figure 2d demonstrates the survey XPS spectrum of RFS and MBC in the binding energy range of 0~1250 eV. The prominent peaks located on 94, 285, 347, 399, 531, 711, 1021, and 1304 eV in RFS are observed, corresponding to Si_2p_, C_1s_, Ca_2p_, N_1s_, O_1s_, Fe_2p_, Zn_2p_, and Mg_1s_, respectively. This result confirms the coexistence of Si, C, Ca, N, O, Fe, Zn, and Mg theoretically [19]. In the high-resolution Fe_2p_ spectrum of RFS and MBC, the peaks of Fe_2p_ at 724.5 and 712.6 eV can be attributed to Fe(III), whilst the enhanced signal at 710.4 eV can be assigned to Fe(II), indicating the partial reduction during the preparation of MBC significantly (Figure 3a,b) [20,21]. For RFS, the peak of C_1s_ at 284.6 eV indicates that it mainly exists in C-H, C-C and C=C (Figure 3c,e). The signal of O at 531.1 eV means that O mainly exists in the form of O-M with Ca, Mg and other metals. However, as for MBC, the peak at 530.3 eV indicates that O also exists in the form of O-C (Figure 3d,f), which proves that the as-prepared MBC is not a simple physical mixing of biochar and Fe_3_O_4_ [21,22].

**Figure 4:**
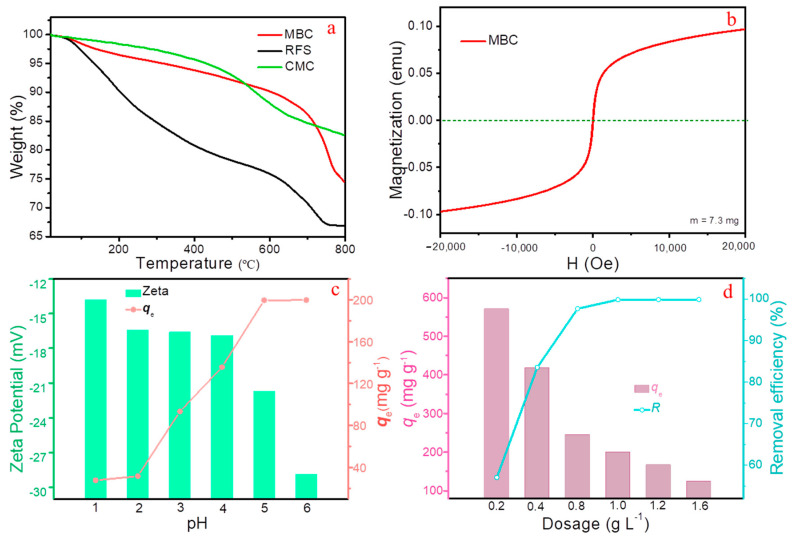
(**a**) TGA curves of the samples, (**b**) Hysteresis loop of MBC, (**c**) Effect of pH and Zeta potential (T = 298 K, C_0_ = 200 mg L^−1^), (**d**) Effect of MBC dosage on Pb(II) adsorption (pH = 5.0, T = 298 K, C_0_ = 200 mg L^−1^).

The thermogravimetric analysis (TGA) technology was employed under N_2_ atmosphere to investigate the thermal stability of RFS, CMC, and MBC (Figure 4a). It can be seen the quality of RFS, CMC, and MBC remains constant without significant quality loss below 120 °C, mainly due to the water evaporation, demonstrating the higher thermal stability. Especially for MBC, good thermal stability below 450 °C fully meets the requirements of conventional adsorption processes, which is also the theoretical basis of selecting 450 °C for the preparation. Considering CMC, the mass loss at temperatures ranging from 120 °C to 600 °C is mainly caused by the decomposition of organic functional groups and cellulose, but the overall loss is within 15%, which means it has about 85% stable mass, being the reason for the stable existence of carbon substrate [23]. Moreover, the specific surface area and pore size distribution of RFS, FS-450, and MBC were further illustrated by N_2_ adsorption-desorption experimental method, as presented in Text S1, Figure S1. Possessing larger specific surface area more appropriate pore size (mesoporous, 2~50 nm) compared to FS-450, that of MBC provides more opportunities for metal ions to reach the surface of adsorbent, thereby enhancing the purification performance of the adsorbent [24,25].

Aiming to fully utilize the abundant iron source of RFS, accompanied by mixing with CMC Na and firing in the tube furnace, high valent iron is partially reduced to obtain magnetic biochar decorated with Fe_3_O_4_, thereby endowing biochar with magnetism and achieving magnetic separation performance. The magnetization investigated via vibrating sample magnetometer (VSM) reveals that MBC possesses favorable superparamagnetism with a saturation magnetization value of 13.15 emu g^−1^ at 300 K (Figure 4b), which fully meets the requirements of magnetic separation. The synergistic effect of CMC-450 and FS-450 ensures that MBC has good adsorption effect and excellent magnetic separation performance.

### 2.2. Adsorption Experiments

#### 2.2.1. Effect of pH

Note that the surface charge of adsorbent and the relative distribution of metal cations on the surface of adsorbent are decisively influenced by the pH value. Therefore, the effect of pH on the adsorption capacity of MBC toward Pb(II) under different pH was investigated; In response, zeta potential values of MBC at diverse pH were further carried out (Figure 4c). To avoid the generation of Pb(OH)_2_ precipitation, this study conducted within the pH range of 1.0~6.0 [26,27]. With the increasing of pH, the Pb(II) uptake was increased (from 1.0 to 5.0) and then remain basically unchanged apparently. Meanwhile it can be seen that the surface charge of MBC always is negative within the operating pH range. When the pH is below 2.0, due to the strong competition between H^+^ and Pb(II), it is difficult for Pb(II) to stabilize on the sites, resulting in a lower adsorption capacity. Due to the coordination interaction between the sides of metal ions and MBC, higher pH can prevent competition between H^+^ and Pb(II). Furthermore, higher negative surface charges will cause greater electrostatic attraction, resulting in the intensive increasing uptake when pH > 2.0. Considering the presence of insoluble Pb(OH)_2_, pH = 5.0 was selected as the optimal adsorption condition in the following batch experiments in this work.

#### 2.2.2. Effect of Dosage

The dosage is the decisive factor affecting the quantity of available active sites [28]. Figure 4d shows that increasing the dosage of MBC enhanced the mercury removal under the conditions of pH = 5.0, T = 298 K, and C_0_ = 200 mg L^−1^, and *t* = 60 min, accompanied by a significant decrease for the equilibrium adsorption capacity. This may be due to the fact that an increase of dosage resulted in more available sites, while the amount of Pb(II) remains basically unchanged, resulting in a relatively insufficient amount of Pb(II) toward per unit mass of adsorbent. Upon continuous increasing the dosage of MBC exceeding 1.0 g L^−1^, the removal efficiencies remain relatively unchanged, whilst the capacities decrease sharply. This may be due to the excessive sites available for Pb(II). To realize a balance, an optimized dosage of 1.0 g L^−1^ was selected in this study.

#### 2.2.3. Adsorption Kinetics

**Figure 5:**
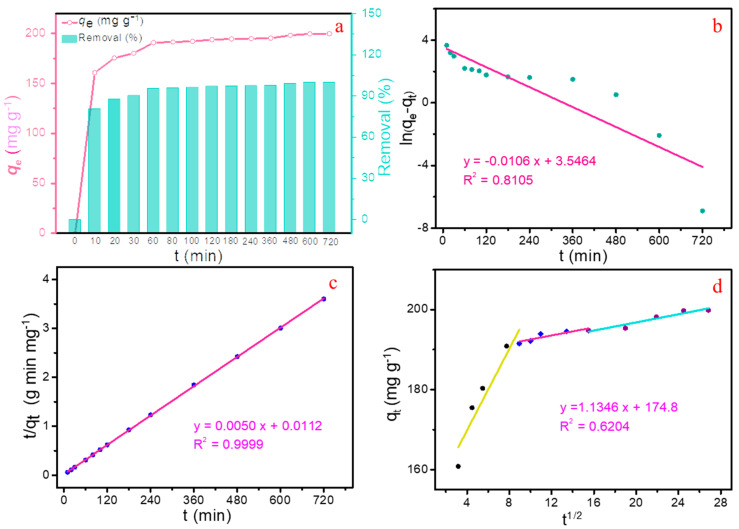
(**a**) Effect of contact time on Pb(II) uptake onto MBC; The fitting of the pseudo-second-order kinetic model (**b**), the pseudo-first-order (**c**) and the intra-particle kinetic models (**d**) for Pb(II) adsorption onto MBC (pH = 5, T = 298 K, C_0_ = 200 mg L^−1^).

The effect of contact time on the adsorption capacity and removal efficiency of Pb(II) is shown in Figure 5a. The Pb(II) was adsorbed onto MBC rapidly in the initial 60 min, and the goals of higher equilibrium adsorption capacity of 190.9 mg g^−1^ and the higher removal rate of 95.5% were achieved simultaneously. Subsequently, as the contact time progressed from 60 min to 720 min, the adsorption amount of Pb(II) only increased from 190.9 mg g^−1^ to 199.9 mg g^−1^, and the corresponding removal rate increased from only 95.5% to 99.9%, maintaining at a quite high level. Therefore, this study selected a contact time of 60 min for the following experiments.

To further insight into the laws of adsorption behavior and the key steps that affect adsorption, three classical kinetic models, including the pseudo-first-order, the pseudo-second-order and the intra-particle kinetic models (Text S2). The final fitting degree between the experimental data and model-predicted result is assessed by the correlation coefficient (*R*^2^). The relevant plots of ln(*q_e_* − *q_t_*) vs. *t*, *t*/*q_t_* vs. *t* and *q_t_* vs. *t*^1/2^ are displayed in Figure 5b–d, and the kinetic parameters are listed in Table 1.

The *R*^2^ value simulated by pseudo-second-order equation is 0.9998, which is significantly higher than that simulated by the other two equations. Furthermore, the data *q_e,exp_* calculated by the pseudo-second-order equation at 298 K is 200.4 mg g^−1^ for Pb(II), being more consistent with the experimental results *q_e,cal_* of 199.9 mg g^−1^ as compared to that of the other models (Table 2). Consequently, the adsorption kinetic of Pb(II) onto MBC fit well with the pseudo-second-order equation, demonstrating that the interaction between Pb(II) and MBC is mainly governed by chemical adsorption [29,30]. The results of nonlinear fitting (Figure S2a), are basically consistent with the trend of linear fitting. In addition, based on the simulation result of intra-particle kinetic model, the process of mass transfer has undergone three stages (Figure 5d), including an instantaneous diffusion stage, an interior surface diffusion stage, and an equilibrium stage.

#### 2.2.4. Effect of Initial Pb(II) Concentrations and Adsorption Isotherms

**Figure 6:**
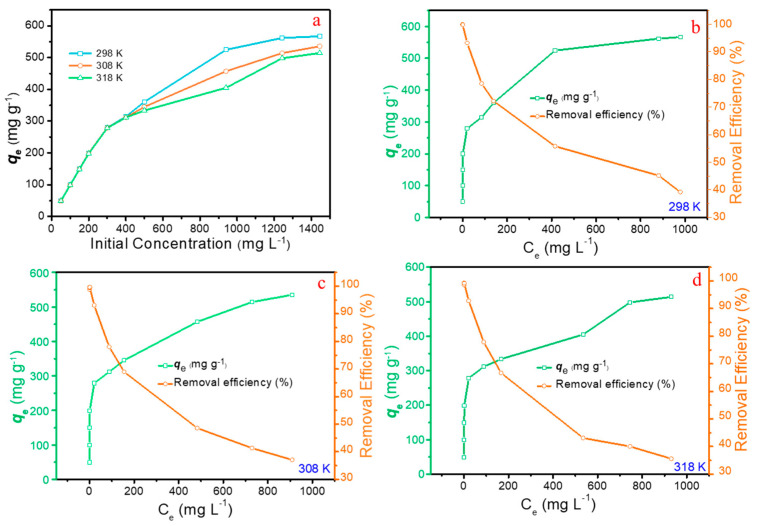
(**a**) Adsorption capacities (*q_e_*) at different temperatures with initial Pb(II) concentrations from 50 to 1445 mg L^−1^; Adsorption isotherm and removal efficiencies of Pb(II) onto MBC of (**b**) T = 298 K, (**c**) T = 308 K and (**d**) T = 318 K (pH = 5.0).

To understand the effect of initial concentration on the removal performance of Pb(II) on MBC, the effect of initial Pb(II) concentrations on removal efficiency is shown in Figure 6a. Over the concentrations (ranging from 50 to 1445 mg L^−1^), the adsorption capacity increases significantly with the increasing initial concentration of Pb(II). This may be attributed to the driving force provided by high concentrations of metal ions, which can better overcome the mass transfer resistance from the solution to the solid phase.

**Figure 7:**
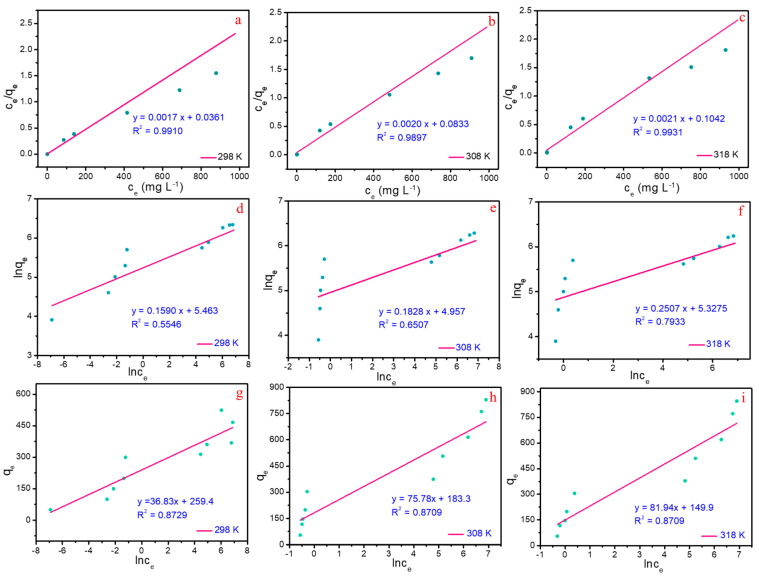
Fitting of Pb(II) adsorption onto MBC (pH = 5.0): The Langmuir model at T = 298 K (**a**), T = 308 K (**b**) and T = 318 K (**c**); the Freundlich isotherm model at T = 298 K (**d**), T = 308 K (**e**) and T = 318 K (**f**); the Temkin isotherm model at T = 298 K (**g**), T = 308 K (**h**) and T = 318 K (**i**).

Typical isotherm models, including the Langmuir, the Freundlich, and the Temkin isotherm models were employed to describe the adsorption equilibrium (Text S3). The plots of *c_e_/q_e_* vs. *c_e_*, ln*q_e_* vs. ln*c_e_* and *q_e_* vs. ln*q_e_* are shown in Figure 7a–i, and the corresponding parameters calculated from the mentioned isotherm models are presented in Table 3. Obviously, the values of *R*^2^ derived from the Langmuir isotherm model stays higher than that calculated by other models, suggesting the data matches the Langmuir isotherm model perfectly. Consequently, this indicates that the adsorption process of Pb(II) on MBC can be classified as the monolayer adsorption with typical adsorption sites. Furthermore, the maximum adsorption capacity *q_m_* simulated via Langmuir isotherm model is 570.7 mg g^−1^ for Pb(II), which is obviously superior to the recently reported magnetic materials (Table S1). The results of nonlinear fitting (Figure S2b–d), are basically consistent with the trend of linear fitting. In addition, excellent adsorption performance in low concentration solutions has been achieved (Figure 6b–d), indicating that MBC also has excellent application prospect in the purification of wastewater with low-concentration Pb(II).

#### 2.2.5. Effect of Temperature and Thermodynamic Analysis

To explore the effect of temperature on the adsorption of Pb(II) onto MBC, the detailed thermodynamic analysis based on the thermodynamic behaviors at 298 K, 308 K, and 318 K is shown in Figure 6a. It can be observed obviously that there is no significant difference among the uptakes at the temperature varying from 298 K to 318 K. when the concentration of Pb(II) is below 400 mg L^−1^. Nevertheless, the adsorption capacities with the concentration exceeding 500 mg L^−1^ were significantly improved at lower temperatures. This phenomenon demonstrates that lower temperature is more favorable for increasing the uptake of Pb(II), which indicating that the adsorption process of Pb(II) onto MBC belongs to an exothermic process.

Furthermore, the thermodynamic parameters, including the standard free-energy change Δ*G*, enthalpy change Δ*H* and entropy change Δ*S* calculated according to the reported literature [31], were listed Table 4. All negative Δ*G* and the decreasing along the increasing temperature fully confirm the spontaneity of adsorption process, and the negative Δ*H* shows that the adsorption belong to a exothermic reaction, and the negative value of Δ*S* verifies the decreasing randomness on the interface between Pb(II) and MBC during the process, indicating that there is basically no change on the biochar during the adsorption [17,32].

#### 2.2.6. Effect of Coexisting Ions

**Figure 8:**
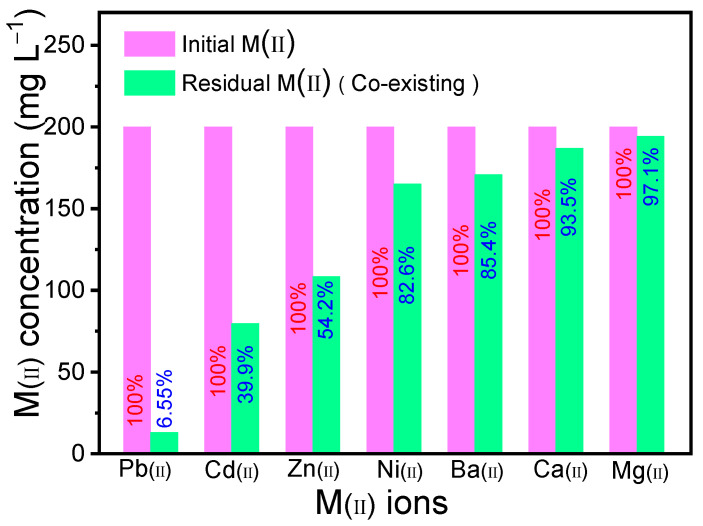
Co-existing heavy metal ions removal performance from a mixed solution of heavy metal ions (pH = 5.0, T = 298 K, C_0_ = 200 mg g^−1^).

The fact that typical divalent metal ions, such as Cd(II), Cu(II), Zn(II), and Mg(II), etc., possibly compete with Pb(II) for binding the available sites on the surface of MBC might result in the decrease of uptake capacity. Competitive adsorption experiments on the interference of coexisting metal ions were conducted, and the results are shown in Figure 8. It can be seen that in the presence of competitive divalent cations, MBC still exhibits higher removal efficiency of 93.45% for Pb(II), indicating that MBC has high selectivity for Pb(II). A smaller hydration radius (4.01 Å) of Pb(II) makes it easier to be complexed or adsorbed by biochar through the inner surface. In addition, as one kind of classic Lewis hard acids, Pb(II) is more easily bound to organic functional groups on biochar [32].

### 2.3. Comparison with the Relevant Adsorbents

The comparison of removal effect between MBC and the recent newly reported materials was conducted. Furthermore, the same adsorption experiments were performed by using some commercial adsorbents to investigate the performance of MBC in practical scenarios, including aluminum oxide, activated carbon, D402 resin, and D401 resin. The performance of adsorption capacity for Pb(II) is shown in Table 5. Obviously, with respect to the adsorption ability of Pb(II) onto the adsorbent, MBC offers markedly superior performance than that of the reported materials and the popular commercial adsorbents, verifying the excellent uptake capability of the as-synthesized MBC.

### 2.4. Plausible Mechanism for Pb(II) Removal Performance

To get insights into the mechanism of adsorption, characterization methods of FT-IR, XRD, XPS, etc., were employed. Furthermore, the peaks of Pb(II) existing in the adsorbent after adsorption confirm the interaction between Pb(II) and MBC (Figure 2d). Combining of these experimental results mentioned, the mechanism for strong removal ability toward Pb(II) can be speculated to the following:

(1) The FT-IR spectra (Figure 2a) verify the presence of oxygen-containing functional groups, such as O-H (3300 cm^−1^), C=O/C=C (1660 cm^−1^), C-N (1415 cm^−1^), and aromatic functional groups (870 cm^−1^) in MBC. Simultaneously, the results of XPS also confirms that in MBC, the percentage of C atoms is 56.8%, mainly existing in the form of C-H, C-C, and C=C; Peaks centered at 531.6 eV and 530.3 eV indicate that O exists in the form of O-C and O-M (M representing metal), respectively. Being classic Lewis hard acids, Pb(II) is more easily complexed with the organic functional groups on MBC [32]. Therefore, the existence of organic functional groups and O-M contained in MBC provide the possibility of efficient adsorption toward Pb(II).

(2) The electrostatic attraction between negatively charged sites on the surface of MBC and positively charged Pb(II), as well as the ion exchange between Pb(II) and O-M, may also be the reason that synthesized MBC can adsorb Pb(II) efficiently.

(3) The SEM results indicate that CMC-450 exhibits a honeycomb-like pore structure with a pore size of 200–600 nm, being the main component of MBC (Figure 2). The bare FS-450 with a diameter of 100–300 nm displays nanoparticles distributed on the carbon thin layer matrix material to form MBC, having a larger specific surface area of 23.08 m^2^ g^−1^. In addition, the morphological characterizations of SEM imply the average pore size of MBC is about 15 nm, and Pb(II) has a smaller hydration radius (4.01 Å), which make it easier for Pb(II) to enter the inner surface of MBC.

## 3. Experimental

### 3.1. Materials and Characterization

All of the reagents used in the experiment were of analytical grade were commercially obtained without any further purification. The detailed chemicals and characterizations are presented in Supplementary Material (Text S4).

### 3.2. Pretreatment of Fenton Sludge (FS)

The Fenton sludge used in this study was taken from the advanced oxidation section of a wastewater treatment plant in an antibiotic pharmaceutical factory in Zhumadian City of Henan Province. The preparation process can be described as following: the paste-like Fenton sludge is filtered using a vacuum filtration system, and the filter residue is obtained. The dewatered sludge was vacuum is dried at 105 °C for 48 h to obtain yellow brown block. Subsequently, the obtained yellow brown blocks are successfully crushed into coarse particles using hammer, grinded in a ball mill, screened with 200-mesh screen, and ultimately dried at 105 °C overnight. The Fenton raw powder (denoted as RFS) is obtained for further use.

The main components of RFS were investigated by XPS, and the results are shown in Table 6. It can be seen that the main elements in RFS include C, N, O, Si, Ca, Fe, etc.

### 3.3. Synthesis of MBC

Take 1.0 g RFS, an appropriate amount of CMC, and 30 mL of water were added into a dry small beaker, stirred magnetically at room temperature for 2 h. Then the suspension was centrifuged, dried, and grounded to obtain mixed powder. The resulting powder was transferred to a quartz boat, wrapped with tin foil, and heated with an increment of 10 °C min^−1^ from room temperature to 450 °C under 100 mL min^−1^ N_2_ (99.9%) in an electric tubular furnace, consistently keeping at 450 °C for 1 h. Ultimately, the resulting sample was cooled to room temperature in an inert atmosphere (99.9% N_2_) before the biochar collection. The obtained power was washed to neutral with hot water, and dried at 80 °C overnight in vacuum oven, which is a novelty magnetic biochar named MBC. Without adding CMC, by the way mentioned above, the black powder obtained is the pyrolysis product of pure RFS, denoted as FS-450. Without adding RFS, the black powder obtained through the same step is the biochar drived from CMC, denoted as CMC-450.

**Figure 9:**
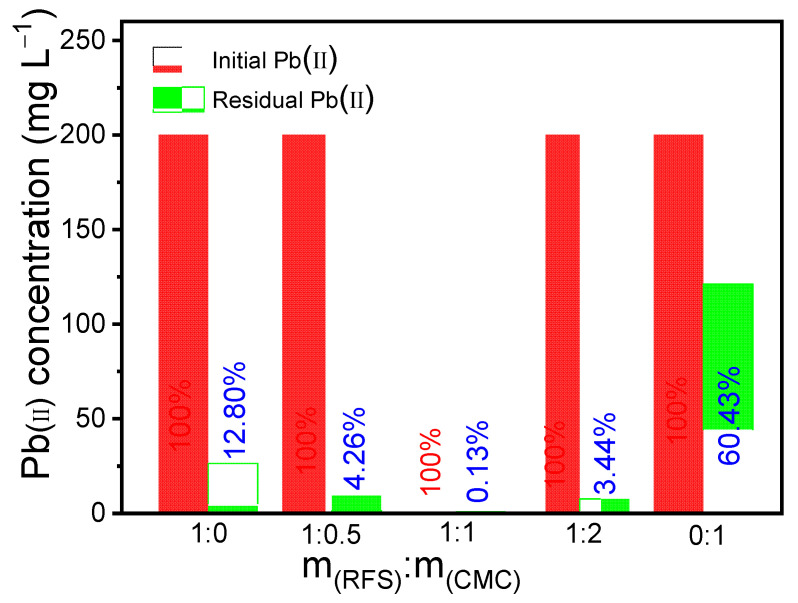
Comparison of adsorption effect under different raw material ratio conditions.

In order to obtain the excellent adsorption performance of MBC for Pb(II), we conducted optimization experiments on the ratio of RFS to CMC. The preparation of MBC with the FRS:CMC mass ratio of 1:0, 1:0.5, 1:1, 1:2, and 0:1 were carried out accordingly, and the adsorption performance of MBC on Pb(II) in simulated water was investigated under the same experimental conditions. The results are shown in Figure 9. It is obviously that the prepared MBC with the mass ratio of 1:1 (FRS:CMC) shows the best adsorption effect toward Pb(II) with a removal rate of up to 99.87%. Therefore, the MBC used in this study were prepared in a mass ratio of 1:1 (FRS: CMC).

### 3.4. Batch Adsorption Experiments

A series of batch experiments were executed to determine the optimum trial condition. For batch adsorption procedures, 25 mg adsorbent was dispersed into 50 mL flask iodine with 25 mL desired Pb(II) solution, and the suspension was shaken at 190 rpm at the desired temperature in thermostatic water bath oscillator. Finally, the adsorbent was separated from the mixing solution via a permanent magnet or filters, and the concentrations of residual metal were detected by inductively coupled plasma-mass spectrometry (ICP-MS). The adsorption capacity *q_e_* (mg g^−1^) and the removal efficiency R (%) were calculated according to the specific formulas (Text S5).

## 4. Conclusions

In summary, a novel magnetic biochar MBC derived from Fenton sludge solid waste and carboxymethyl cellulose sodium was successfully fabricated via a one-step pyrolysis method, which reuses the solid waste resource, reduces the cost of MBC, and simplifies the preparation process simultaneously. Detailed characterizations verify the formation of the MBC composite. The as-prepared MBC exhibited superior adsorption ability toward Pb(II) (570.7 mg g^−1^) at optimized operating parameters, affording excellent selectivity, easy separation and environmental friendliness. The adsorption performances were well-fitted with the pseudo-second-order model, Langmuir isotherm model, and spontaneous endothermic process. Additionally, the adsorption mechanism is investigated thoroughly with the aid of FT-IR, XPS, and SEM analysis and batch experimental trials. All results demonstrate that MBC holds great promise in application for the remediation of Pb(II)-contaminated wastewater.

## Figures and Tables

**Table 1:** Kinetic parameters for Pb(II) adsorption onto MBC (T = 298 K, C_0_ = 200 mg L^−1^).

Kinetic Model	Parameter	Value	*R* ^2^
Pseudo-first-order	*k*_1_ (min^−1^)	3.546	0.8105
	*q_e_* (mg g^−1^)	34.68	
Pseudo-second-order	*k*_2_ (mg min^−1^ g^−1^)	2.2 × 10^−3^	0.9999
	*q_e_* (mg g^−1^)	200.4	
Intraparticle diffusion	*k*_dif_ (mg g^−1^)	1.134	0.6205
	C (mg g^−1^ min^−1^)	174.8	

**Table 2:** Relevant data of adsorption for Pb(II) onto MBC (T = 298 K, pH = 5.0, C_0_ = 200 mg L^−1^).

Ion	*q_e,exp_* (mg g^−1^)	*q_e,cal_* (mg g^−1^)
Pb(II)	199.9	200.4

**Table 3:** Isotherm parameters for Pb(II) adsorption onto MBC (T = 298 K, pH = 5.0).

Adsorption Isotherm	Parameter	Value			*R* ^2^		
		298 K	308 K	318 K	298 K	308 K	318 K
Langmuir	*q_m_* (mg g^−1^)	570.7	508.0	470.7	0.9954	0.9948	0.9965
	*K_L_* (L g^−1^)	0.0485	0.0236	0.0204			
Freundlich	*K_F_* (mg g^−1^)	235.9	142.2	129.9	0.5546	0.6507	0.8139
	*n*	6.288	5.472	5.266			
Temkin	*b_T_*	67.26	32.69	30.08	0.9343	0.9332	0.9411
	*K_T_*	1144	11.23	6.19			

**Table 4:** Thermodynamic parameters for Pb(II) adsorption onto MBC at different temperatures.

Δ*G* (kJ mol^−1^)			Δ*H* (kJ mol^−1^)	Δ*S* (J mol^−1^ K^−1^)
298 K	308 K	318 K		
−16.29	−14.93	−13.59	−56.50	−134.3

**Table 5:** Comparison of adsorption capacity for Pb(II) onto MBC with the relevant adsorbents.

Adsorbent	C_0_ (mg L^−1^)	*q_e_* (mg g^−1^)	Ref.
CS-biochar-500	200	165.0	[5]
MPH-220	200	110.9	[4]
HP-BC	300	43.6	[33]
MgFe_2_O_4_–NH_2_@sRHB	500	199.1	[34]
Aluminum oxide	400	20.6	This study
Activated carbon	400	47.3	This study
D402 resin	400	238.9	This study
D401 resin	400	146.9	This study
MBC	400	314.3	This study

**Table 6:** The main components of RFS and MBC.

Element	RFS (Atom %)	MBC (Atom %)
C	30.05	56.8
N	1.53	2.44
O	43.57	27.72
Si	13.32	3.72
Ca	3.99	2.97
Fe	6.70	4.24
Mg	0.73	1.29
Zn	0.12	0.82

## Supplementary Materials

The following supporting information can be downloaded at: https://www.mdpi.com/article/10.3390/molecules28134983/s1. Magnetic biochar derived from Fenton sludge/CMC for high-efficiency removal of Pb(II):synthesis, application, and mechanism. References [24,25,35,36,37,38,39,40,41,42,43,44,45,46] are cited in Supplementary Materials.
