# Supplementary material for: Magnetic Biochar Derived from Fenton Sludge/CMC for High-Efficiency Removal of Pb(II): Synthesis, Application, and Mechanism
Source: Molecules. 2023 Jun 25;28(13):4983. doi: 10.3390/molecules28134983 (PMC10343431; doi:10.3390/molecules28134983)
Supplement: Supplementary file 1 [file molecules-28-04983-s001.zip › molecules-2426586-supplementary.pdf]

Magnetic biochar derived from Fenton sludge/CMC for high-efficiency removal of Pb(II):synthesis, application, and mechanism

Zongwu Wang[1,2], Juan Guo[1],Junwei Jia[1], Wei Liu[1], Xinding Yao[1], Jinglan Feng[2], Shuying Dong[2,*], Jianhui Sun[2,*]

[1]*Department of Environment Engineering, Yellow River Conservancy Technical Institute, Kaifeng Engineering Research Center for Municipal Wastewater Treatment, Kaifeng, 475004, P. R. China*

[2]*MOE Key Laboratory of Yellow River and Huai River Water Environmental and Pollution Control, School of Environment, Henan Normal University, Xinxiang, Henan, 453007, P. R. China*

*Corresponding authors. E-mail: sunjh@htu.edu.cn (J. Sun); dongsy@htu.edu.cn (S. Dong)

Total number of pages of Supporting Information: **8**

Number of Texts in Supporting Information: **5**

Number of Figures in Supporting Information:**2**

Number of Tables in Supporting Information:**1**

List of the Supplemental Texts

Text S1. The results of N_2 adsorption–desorption.
Text S2. Kinetics models
Text S3. Isotherm models
Text S4. Materials and Instrumentation
Text S5. The formulas of adsorption capacity q_e (mg g^{-1}) and the removal efficiency R (%)

List of the Supplemental Figures

Figure S1. BET analysis of the samples.

Figure S2. The nonlinear fitting of the pseudo-second-order kinetic model, the pseudo-first-order and the intra-particle kinetic models (a) for Pb(II) adsorption onto MBC (pH = 5, T = 298 K, C_0 = 200 mg L^{-1}); The nonlinear itting of Pb(II) adsorption onto MBC (pH = 5.0): the Langmuir, Freundlich isotherm and Temkin isotherm model at T = 298 K (b), T = 308 K (c) and T = 318 K(d).

List of the Supplemental Tables

Table S1. Comparison of Pb(II) uptake by various magnetic adsorbents.

Text S1

The specific surface area and pore size distribution of RFS, FS-450, and MBC were measured using N_2 adsorption desorption experimental method, and the correspodngi results are shown in Fig. S1. The specific surface area of FS-450 is 15.24 m^2 g^{-1}, while that of MBC is 23.08 m^2 g^{-1}(Fig. S1a). This indicates that compared with a single FS-450, MBC not only utilizes the rich iron source of RFS to obtain magnetic substances, but also has an increase in specific surface area, which helps to improve the adsorption capacity of MBC. In addition, the average pore sizes of RFS and FS-450 measured are 5.96 nm and 30 nm respectively, while the average pore size of MBC is 15 nm(Fig. S1b,c), indicating that the pores in MBC samples mainly exist in the form of mesopores. From Fig. S1a, it can be seen that there is significant adsorption at higher relative pressures (P/P0>0.8), indicating the presence of mesopores in the skeleton, which is consistent with the pore size measurement results mentioned above [1]. MBC has a relatively large specific surface area; Compared with FS-450, the appropriate pore size (mesoporous, 2-50 nm) of MBC provides more opportunities for metal ions to reach the surface of the composite material, thereby enhancing the purification performance of the adsorbent [2, 3].

Figure S1

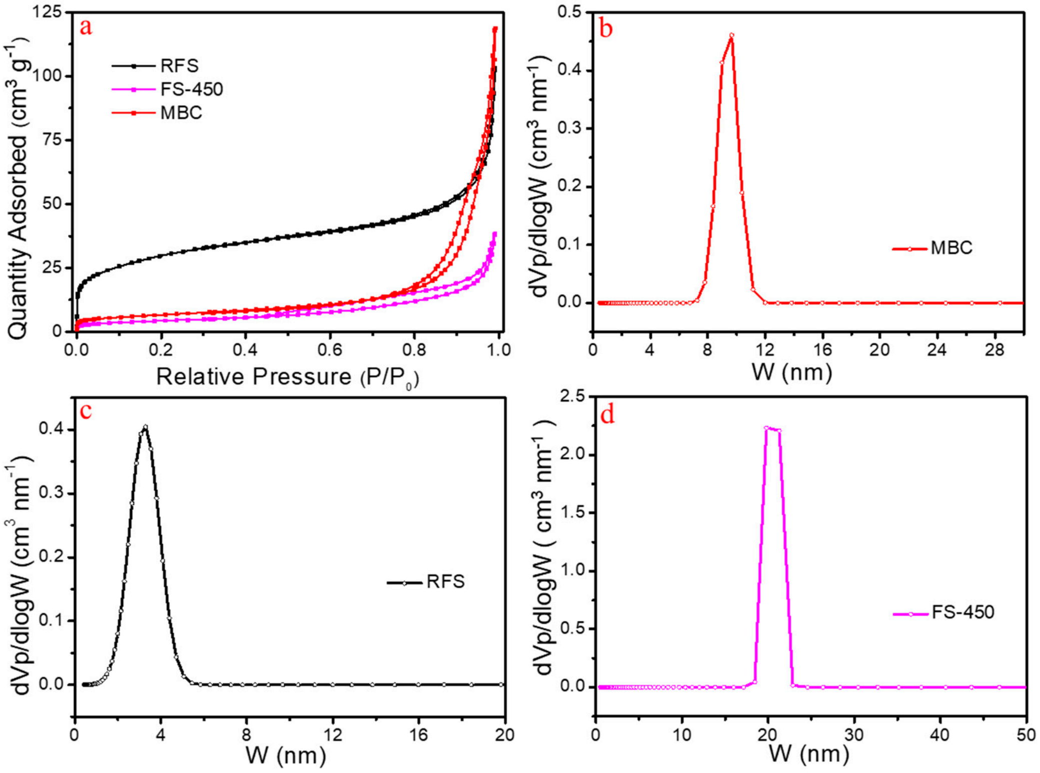

Figure S1. BET analysis of the samples (a); Distribution of pore diameter (b) MBC, (c) RFS and (d) FS-450.

Text S2

The pseudo-first-order, the pseudo-second-order equation and the intra-particle kinetic model, which are expressed as Eqs. (3), (4) and (5), respectively.

$$\ln(q_e - q_t) = \ln q_e - k_1 t \quad \text{or} \quad q_t = q_e(1 - \exp(-k_1 t)) \tag{3}$$

$$\frac{t}{q_t} = \frac{1}{k_2 q_e^2} + \frac{t}{q_e} \quad \text{or} \quad q_t = \frac{t}{(1/k_2 q_e^{\ 2} + t/q_e)} \tag{4}$$

$$q_t = k_{dif} t^{1/2} + C \tag{5}$$

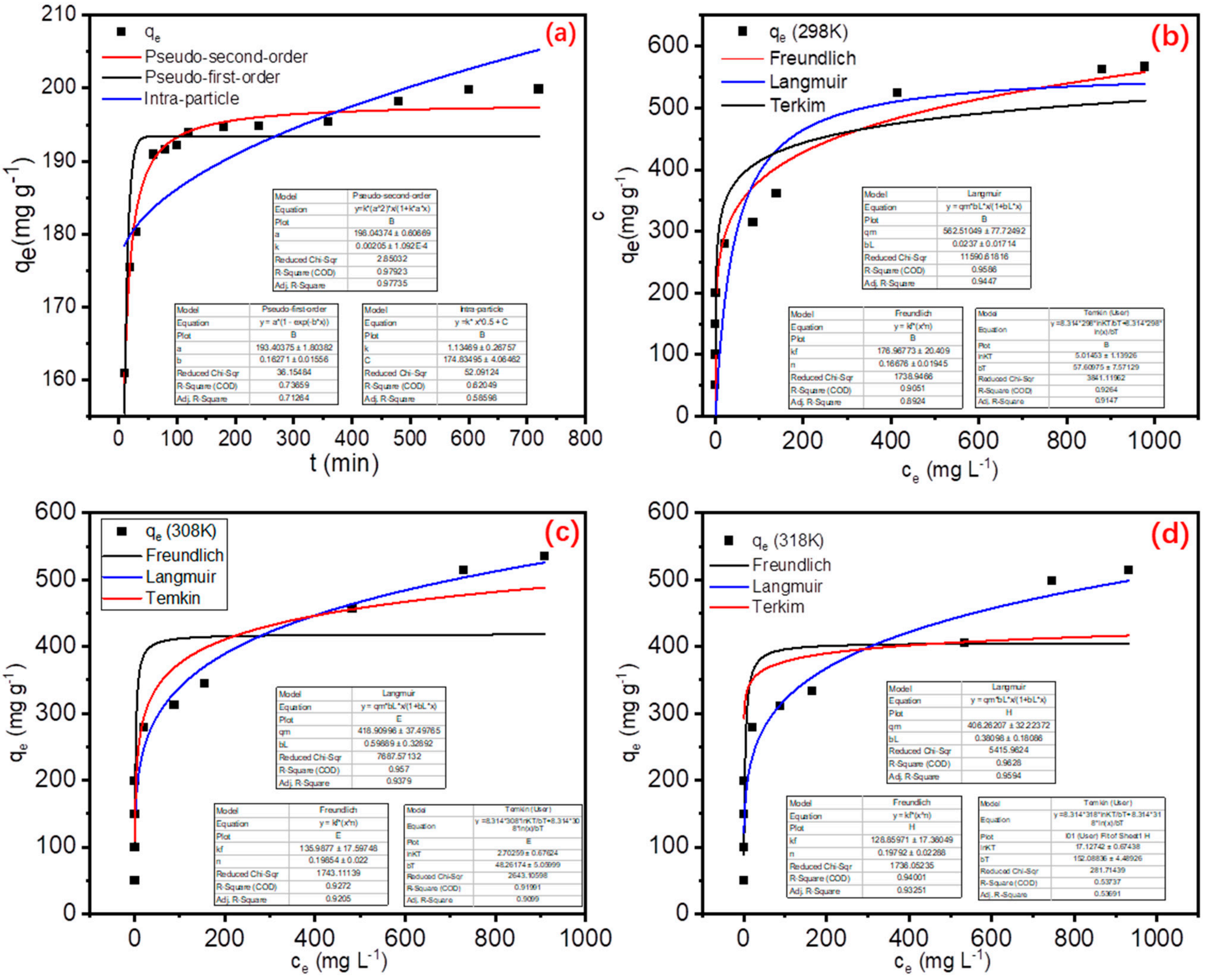

Figure S2. The nonlinear fitting of the pseudo-second-order kinetic model, the pseudo-first-order and the intra-particle kinetic models (a) for Pb(II) adsorption onto MBC (pH = 5, T = 298 K, C_0 = 200 mg L^{-1}); The nonlinear itting of Pb(II) adsorption onto MBC (pH = 5.0): the Langmuir, Freundlich isotherm and Temkin isotherm model at T = 298 K (b), T = 308 K (c) and T = 318 K(d).

Text S3

The Langmuir isotherm model, Freundlich isotherm model and Temkin isotherm model.

$$\frac{c_e}{q_e} = \frac{c_e}{q_m} + \frac{1}{q_m K_L} \quad \text{or} \quad q_e = \frac{q_m K_L c_e}{1 + K_L c_e} \tag{5}$$

$$\ln q_e = \frac{1}{n} \ln c_e + \ln K_F \quad \text{or} \quad q_e = K_F c_e^{1/n} \tag{6}$$

$$q_e = \frac{RT}{b_T} \ln K_T + \frac{RT}{b_T} \ln c_e \tag{7}$$

Table S1

Table S1. Comparison of Pb(II) uptake by various magnetic adsorbents.

Adsorbent	Metal ions	q_{max} (mg g^{-1})	Magnetization (emu g^{-1})	Ref.
CS-PAM-MCM	Pb(II)	63.67	7.6	[4]
Fe_3O_4@SiO_2-EDTA	Pb(II)	113.9	34.49	[5]
MAF-SCMNPs	Pb(II)	292	33.4	[6]
GO/ Fe_3O_4-g-G3.0	Pb(II)	181.4	13.8	[7]
MCGO	Pb(II)	112.35	—	[8]
rGO-PDTC/Fe_3O_4	Pb(II)	147.06	—	[9]
Fe_3O_4@SiO_2-HO-S	Pb(II)	178.02	—	[10]
rGO-p($C_3N_3S_3$)/Fe_3O_4	Pb(II)	270.3	19.5	[11]
β-CD@MRHC	Pb(II)	266.2	17.36	[12]
MP	Pb(II)	139.28	11.51	[13]
$Ni_{0.6}Fe_{2.4}O_4$-HT-COF	Pb(II)	411.80	39.83	[14]
MBC	Pb(II)	570.7	13.15	This study

Text S4

Materials: sodium carboxymethyl cellulose ($[C_6H_7O_2(OH)_2OCH_2COONa]_n$) and lead nitrate ($Pb(NO_3)_2$) and were purchased from Macklin Reagents. Silica gel (60-80 mush) was purchased from Shanghai Institute of Inorganic Chemical Industry. Aluminum oxide was purchased from Shanghai Wusi Farm Chemical Reagent Factory. Activated carbon was purchased from Xinxiang City Pharmaceutical Company. Ion Exchange Resin was purchased from Jiangsu Suqing Water Treatment Engineering Group Co., Ltd. Pure water was obtained from Wahaha Group Co., Ltd.

Instrumentation: X-ray diffraction (XRD) pattern was collected on Bruker D8-AXS. X-ray photoelectron spectrometer (XPS) measurements were recorded on Escalab 250Xi. Structure and morphology were observed by scanning electron microscope (SEM) (SUPRA40, Carl Zeiss) and transmission electron microscopy (TEM) (JEM-2100, JEOL). The thermogravimetric analysis (TGA) was carried out with thermal analyzer (STA449F3, NETZSCH) under N_2 flow from 25℃ to 900℃. N_2 adsorption-desorption isotherms were performed using Micromeritics ASAP2020. Magnetization was obtained using vibrating sample magnetometer (VSM) (MPMS3, Quantum). The pH-dependent surface charge was investigated by Zeta Potential (Mastersizer2000, Malvern).

The concentration of metal ions was determined by inductively coupled plasma-mass spectrometry (ICP-MS, PE ELAN DRC-e).

Text S5

The adsorption capacity q_e (mg g^{-1}) and removal efficiency of adsorbent was calculated according the following equations:

$$q_{\mathrm{e}} = \frac{(C_0 - C_e)V}{m} \tag{1}$$

$$R\% = \frac{C_0 - C_e}{C_0} \times 100\% \tag{2}$$

where V (L) is the volume of the water solution, C_0 and C_e (mg L^{-1}) are the initial and equilibrium concentrations of the metal ions, m (g) is the mass of adsorbent used. All experiments were performed in triplicate.

References

[1] Thomas J. Barton, Lucy M. Bull, Walter G. Klemperer, et al. Tailored Porous Materials[J]. Chemistry of Materials, 1999, 11: 2633-2656.

[2] Dongah Ko, Joo Sung Lee, Hasmukh A. Patel, et al. Selective removal of heavy metal ions by disulfide linked polymer networks[J]. Journal of Hazardous Materials, 2017, 332: 140-148.

[3] K.s.w. Sing, D.h. Everett, R.a.w. Haul, et al. Reporting physisorption data for gas/solid syste-ms with special reference to the determination of surface area and porosity[J]. Pure and Applied Chemis-try , 1985, 57: 603-619.

[4] Li K., Wang Y.W., Huang M., Yan H., 2015. Preparation of chitosan-graft-polyacrylamide magnetic composite microspheres for enhanced selective removal of mercury ions from water. J. Colloid and Interf.Sci. 455, 261-270.

[5] Liu Y., Fu R.Q., Sun Y., 2016. Multifunctional nanocomposites Fe3O4@SiO2-EDTA for Pb(II) and Cu(II) removal from aqueous solutions. Appl Surf Sci 369, 267-276.

[6] Bao S.Y., Li K., Ning P., Peng j.H., 2017. Highly effective removal of mercury and lead ions from wastewater by mercaptoamine-functionalised silica-coated magnetic nano-adsorbents: Behaviours and mechanisms. Appl. Surf. Sci. 393, 457-466.

[7] Ma Y.X., Kou Y.L., Xing D., 2017. Synthesis of magnetic graphene oxide grafted polymaleicamide dendrimer nanohybrids for adsorption of Pb(II) in aqueous solution. Journal of hazardous materials 340, 407-416.

[8] Samuel M.S., Shah S.S., Bhattacharya J., 2018. Adsorption of Pb(II) from aqueous solution using a magnetic chitosan/graphene oxide composite and its toxicity studies. International journal of biological macromolecules 115, 1142-1150.

[9] Fu W., Huang Z.Q., 2018. Magnetic dithiocarbamate functionalized reduced graphene oxide for the removal of Cu(II), Cd(II), Pb(II), and Hg(II) ions from aqueous solution: Synthesis, adsorption, and regeneration. Chemosphere 209, 449-456.

[10] Zhao J.J., Niu Y.Z., Ren B., Chen H., 2018. Synthesis of Schiff base functionalized superparamagnetic Fe3O4

composites for effective removal of Pb(II) and Cd(II) from aqueous solution. Chem. Eng. J. 347, 574-584.
[11] Fu W., Wang X.Y., Huang Z.Q., 2019. Remarkable reusability of magnetic Fe3O4-encapsulated C3N3S3 polymer/reduced graphene oxide composite: A highly effective adsorbent for Pb and Hg ions. Sci.Total Environ. 659, 895-904.
[12] Liu J , Zhou J , Wu Z , et al. Concurrent elimination and stepwise recovery of Pb(Ⅱ) and bisphenol A from water using β-cyclodextrin modified magnetic cellulose: adsorption performance and mechanism investigation[J]. Journal of hazardous materials, 2022(Jun.15):432.
[13] Li R , Lan G , Liu Y . Melamine sponge loading improves the separation performance of magnetic hydroxy apatite for Pb(II) adsorption[J]. Separation and Purification Technology, 2022,291: 120851.
[14] Shuai Wang, Hao Wang. Novel magnetic covalent organic framework for the selective and effective removal of hazardous metal Pb(II) from solution: Synthesis and adsorption characteristics[J]. Separation and Purification Technology, 2023,307: 122783
